# Supplementary material for: Meta-Analysis and Gene Set Enrichment Relative to ER Status Reveal Elevated Activity of MYC and E2F in the “Basal” Breast Cancer Subgroup
Source: PLoS One. 2009 Mar 9;4(3):e4710. doi: 10.1371/journal.pone.0004710 (PMC2650420; doi:10.1371/journal.pone.0004710)
Supplement: Dataset S1 — GSEA results from first screen. The file contains a zipped archive of the HTML results of the second GSEA screen. Reports for enriched or depleted datasets may be accessed from the “index.html” link within the my_analysis.GseaPreranked.1219975950625.rpt.zip ZIP archive. Enrichment plots are available for each of the 50 most enriched and depleted gene sets. (13.32 MB ZIP) [file pone.0004710.s001.zip › my_analysis.GseaPreranked.1219975950625.rpt.zip!//gsea_report_for_na_neg_1219975950625.html]

Report for na\_neg 1219975950625 [GSEA]

| GS  follow link to MSigDB | GS DETAILS | SIZE | ES | NES | NOM p-val | FDR q-val | FWER p-val | RANK AT MAX | LEADING EDGE || 1 | BRCA\_ER\_POS | Details ... | 478 | -0.79 | -4.40 | 0.000 | 0.000 | 0.000 | 1925 | tags=72%, list=14%, signal=81% |
| 2 | BRCA\_BRCA1\_NEG | Details ... | 140 | -0.70 | -3.40 | 0.000 | 0.000 | 0.000 | 2337 | tags=60%, list=18%, signal=72% |
| 3 | BRCA\_PROGNOSIS\_POS | Details ... | 38 | -0.80 | -2.99 | 0.000 | 0.000 | 0.000 | 1232 | tags=66%, list=9%, signal=72% |
| 4 | VANTVEER\_BREAST\_OUTCOME\_GOOD\_VS\_POOR\_UP | Details ... | 25 | -0.83 | -2.82 | 0.000 | 0.000 | 0.000 | 1180 | tags=72%, list=9%, signal=79% |
| 5 | AGUIRRE\_PANCREAS\_CHR12 | Details ... | 59 | -0.58 | -2.43 | 0.000 | 0.000 | 0.000 | 2267 | tags=53%, list=17%, signal=63% |
| 6 | CHR3P21 | Details ... | 167 | -0.49 | -2.41 | 0.000 | 0.000 | 0.000 | 2768 | tags=47%, list=21%, signal=58% |
| 7 | CHR16P13 | Details ... | 174 | -0.48 | -2.41 | 0.000 | 0.000 | 0.000 | 2576 | tags=41%, list=19%, signal=51% |
| 8 | CHR14Q11 | Details ... | 86 | -0.51 | -2.33 | 0.000 | 0.000 | 0.001 | 1877 | tags=40%, list=14%, signal=46% |
| 9 | FRASOR\_ER\_UP | Details ... | 30 | -0.63 | -2.28 | 0.000 | 0.000 | 0.001 | 933 | tags=43%, list=7%, signal=46% |
| 10 | CHR12Q13 | Details ... | 155 | -0.45 | -2.27 | 0.000 | 0.000 | 0.004 | 2052 | tags=34%, list=15%, signal=39% |
| 11 | CHR5Q13 | Details ... | 30 | -0.62 | -2.25 | 0.000 | 0.000 | 0.007 | 1807 | tags=47%, list=14%, signal=54% |
| 12 | AGUIRRE\_PANCREAS\_CHR17 | Details ... | 70 | -0.51 | -2.22 | 0.000 | 0.001 | 0.009 | 2974 | tags=49%, list=22%, signal=62% |
| 13 | CHR11Q13 | Details ... | 183 | -0.42 | -2.08 | 0.000 | 0.005 | 0.081 | 2920 | tags=45%, list=22%, signal=57% |
| 14 | CHR5Q11 | Details ... | 22 | -0.61 | -2.04 | 0.000 | 0.007 | 0.133 | 2295 | tags=50%, list=17%, signal=60% |
| 15 | HDACI\_COLON\_TSA2HRS\_UP | Details ... | 49 | -0.51 | -2.03 | 0.000 | 0.008 | 0.148 | 1813 | tags=39%, list=14%, signal=45% |
| 16 | CHR17Q24 | Details ... | 26 | -0.59 | -2.02 | 0.003 | 0.008 | 0.167 | 2063 | tags=35%, list=15%, signal=41% |
| 17 | CHR5Q31 | Details ... | 128 | -0.42 | -2.00 | 0.000 | 0.009 | 0.189 | 2350 | tags=34%, list=18%, signal=40% |
| 18 | CHR16P12 | Details ... | 57 | -0.47 | -1.99 | 0.000 | 0.009 | 0.205 | 974 | tags=25%, list=7%, signal=26% |
| 19 | CHR14Q22 | Details ... | 42 | -0.50 | -1.98 | 0.000 | 0.010 | 0.226 | 2288 | tags=43%, list=17%, signal=52% |
| 20 | CHR5Q22 | Details ... | 15 | -0.66 | -1.97 | 0.002 | 0.011 | 0.252 | 2447 | tags=67%, list=18%, signal=82% |
| 21 | CHR17Q21 | Details ... | 184 | -0.39 | -1.97 | 0.000 | 0.010 | 0.253 | 2541 | tags=34%, list=19%, signal=41% |
| 22 | GH\_AUTOCRINE\_DN | Details ... | 107 | -0.41 | -1.94 | 0.000 | 0.014 | 0.340 | 2468 | tags=43%, list=19%, signal=52% |
| 23 | FRASOR\_ER\_DN | Details ... | 61 | -0.45 | -1.92 | 0.000 | 0.014 | 0.367 | 1630 | tags=36%, list=12%, signal=41% |
| 24 | AGED\_MOUSE\_HIPPOCAMPUS\_ANY\_UP | Details ... | 41 | -0.50 | -1.92 | 0.003 | 0.015 | 0.380 | 2321 | tags=46%, list=17%, signal=56% |
| 25 | PHOTOSYNTHESIS | Details ... | 20 | -0.58 | -1.88 | 0.003 | 0.020 | 0.491 | 2359 | tags=50%, list=18%, signal=61% |
| 26 | CHR14Q23 | Details ... | 25 | -0.53 | -1.85 | 0.000 | 0.027 | 0.602 | 2999 | tags=40%, list=23%, signal=52% |
| 27 | CHR16P11 | Details ... | 59 | -0.45 | -1.84 | 0.000 | 0.028 | 0.629 | 2731 | tags=41%, list=21%, signal=51% |
| 28 | HDACI\_COLON\_BUT12HRS\_UP | Details ... | 38 | -0.49 | -1.84 | 0.000 | 0.028 | 0.642 | 2761 | tags=47%, list=21%, signal=60% |
| 29 | HDACI\_COLON\_TSABUT\_UP | Details ... | 62 | -0.43 | -1.81 | 0.000 | 0.035 | 0.740 | 1533 | tags=31%, list=12%, signal=34% |
| 30 | TYPE\_III\_SECRETION\_SYSTEM | Details ... | 19 | -0.55 | -1.80 | 0.000 | 0.038 | 0.778 | 2359 | tags=47%, list=18%, signal=57% |
| 31 | ADIP\_DIFF\_CLUSTER1 | Details ... | 55 | -0.43 | -1.78 | 0.000 | 0.042 | 0.824 | 1422 | tags=29%, list=11%, signal=32% |
| 32 | FLAGELLAR\_ASSEMBLY | Details ... | 19 | -0.55 | -1.77 | 0.005 | 0.044 | 0.845 | 2359 | tags=47%, list=18%, signal=57% |
| 33 | ATP\_SYNTHESIS | Details ... | 19 | -0.55 | -1.76 | 0.010 | 0.049 | 0.886 | 2359 | tags=47%, list=18%, signal=57% |
| 34 | CHR5Q12 | Details ... | 25 | -0.51 | -1.75 | 0.003 | 0.048 | 0.889 | 1060 | tags=36%, list=8%, signal=39% |
| 35 | HDACI\_COLON\_TSA\_UP | Details ... | 100 | -0.38 | -1.75 | 0.000 | 0.047 | 0.889 | 1813 | tags=31%, list=14%, signal=36% |
| 36 | POMEROY\_MD\_TREATMENT\_GOOD\_VS\_POOR\_UP | Details ... | 29 | -0.48 | -1.74 | 0.003 | 0.053 | 0.924 | 1794 | tags=38%, list=13%, signal=44% |
| 37 | BOQUEST\_CD31PLUS\_VS\_CD31MINUS\_DN | Details ... | 265 | -0.33 | -1.73 | 0.000 | 0.054 | 0.935 | 1512 | tags=24%, list=11%, signal=27% |
| 38 | CHR4P16 | Details ... | 67 | -0.40 | -1.73 | 0.003 | 0.053 | 0.935 | 2009 | tags=31%, list=15%, signal=37% |
| 39 | VERNELL\_PRB\_CLSTR2 | Details ... | 20 | -0.53 | -1.72 | 0.005 | 0.055 | 0.950 | 651 | tags=30%, list=5%, signal=31% |
| 40 | CHR14Q12 | Details ... | 15 | -0.59 | -1.72 | 0.015 | 0.056 | 0.951 | 2186 | tags=40%, list=16%, signal=48% |
| 41 | ET743\_RESIST\_UP | Details ... | 17 | -0.55 | -1.68 | 0.027 | 0.074 | 0.985 | 1448 | tags=41%, list=11%, signal=46% |
| 42 | CGGAARNGGCNG\_UNKNOWN | Details ... | 35 | -0.45 | -1.68 | 0.008 | 0.074 | 0.986 | 2798 | tags=40%, list=21%, signal=51% |
| 43 | GTTNYYNNGGTNA\_UNKNOWN | Details ... | 56 | -0.41 | -1.67 | 0.010 | 0.075 | 0.988 | 2100 | tags=27%, list=16%, signal=32% |
| 44 | BRENTANI\_TRANSPORT\_OF\_VESICLES | Details ... | 23 | -0.48 | -1.63 | 0.016 | 0.108 | 0.998 | 834 | tags=22%, list=6%, signal=23% |
| 45 | TSA\_HEPATOMA\_CANCER\_UP | Details ... | 39 | -0.42 | -1.61 | 0.013 | 0.119 | 0.998 | 2124 | tags=38%, list=16%, signal=46% |
| 46 | KANNAN\_P53\_UP | Details ... | 35 | -0.43 | -1.60 | 0.014 | 0.124 | 0.999 | 2668 | tags=37%, list=20%, signal=46% |
| 47 | CHR17Q23 | Details ... | 51 | -0.39 | -1.59 | 0.003 | 0.131 | 0.999 | 2333 | tags=37%, list=18%, signal=45% |
| 48 | CHR12Q14 | Details ... | 24 | -0.47 | -1.59 | 0.024 | 0.132 | 0.999 | 1631 | tags=25%, list=12%, signal=28% |
| 49 | HDACI\_COLON\_CUR24HRS\_DN | Details ... | 17 | -0.52 | -1.59 | 0.023 | 0.133 | 0.999 | 1686 | tags=47%, list=13%, signal=54% |
| 50 | CHR12Q12 | Details ... | 40 | -0.42 | -1.58 | 0.015 | 0.133 | 0.999 | 1306 | tags=30%, list=10%, signal=33% |
| 51 | HDACI\_COLON\_BUT16HRS\_UP |  | 35 | -0.42 | -1.57 | 0.006 | 0.139 | 1.000 | 1838 | tags=34%, list=14%, signal=40% |
| 52 | CHR11Q14 |  | 28 | -0.46 | -1.56 | 0.019 | 0.147 | 1.000 | 870 | tags=25%, list=7%, signal=27% |
| 53 | RYCACNNRNNRNCAG\_UNKNOWN |  | 47 | -0.39 | -1.56 | 0.015 | 0.147 | 1.000 | 879 | tags=19%, list=7%, signal=20% |
| 54 | AGED\_MOUSE\_HIPPOCAMPUS\_MULTI\_UP |  | 18 | -0.50 | -1.56 | 0.035 | 0.145 | 1.000 | 2321 | tags=50%, list=17%, signal=60% |
| 55 | KRCTCNNNNMANAGC\_UNKNOWN |  | 49 | -0.38 | -1.55 | 0.019 | 0.148 | 1.000 | 2277 | tags=31%, list=17%, signal=37% |
| 56 | ADIP\_HUMAN\_DN |  | 25 | -0.44 | -1.55 | 0.032 | 0.151 | 1.000 | 1871 | tags=36%, list=14%, signal=42% |
| 57 | CHR14Q24 |  | 72 | -0.36 | -1.54 | 0.014 | 0.156 | 1.000 | 1783 | tags=26%, list=13%, signal=30% |
| 58 | CHR4Q35 |  | 19 | -0.48 | -1.52 | 0.034 | 0.180 | 1.000 | 1600 | tags=32%, list=12%, signal=36% |
| 59 | CIS\_RESIST\_GASTRIC\_UP |  | 16 | -0.50 | -1.51 | 0.043 | 0.184 | 1.000 | 2692 | tags=44%, list=20%, signal=55% |
| 60 | CHR20P11 |  | 35 | -0.40 | -1.50 | 0.028 | 0.204 | 1.000 | 2511 | tags=43%, list=19%, signal=53% |
| 61 | CYTAGCAAY\_UNKNOWN |  | 94 | -0.33 | -1.49 | 0.000 | 0.203 | 1.000 | 1476 | tags=23%, list=11%, signal=26% |
| 62 | HDACI\_COLON\_BUT2HRS\_UP |  | 64 | -0.35 | -1.49 | 0.015 | 0.211 | 1.000 | 2973 | tags=39%, list=22%, signal=50% |
| 63 | CHR12Q15 |  | 20 | -0.45 | -1.47 | 0.051 | 0.225 | 1.000 | 1787 | tags=35%, list=13%, signal=40% |
| 64 | CHR4Q32 |  | 23 | -0.44 | -1.47 | 0.053 | 0.228 | 1.000 | 2046 | tags=35%, list=15%, signal=41% |
| 65 | TGFBETA\_C1\_UP |  | 17 | -0.47 | -1.47 | 0.047 | 0.226 | 1.000 | 2124 | tags=47%, list=16%, signal=56% |
| 66 | ALCALAY\_AML\_NPMC\_UP |  | 139 | -0.30 | -1.47 | 0.012 | 0.223 | 1.000 | 2563 | tags=36%, list=19%, signal=44% |
| 67 | HDACI\_COLON\_CLUSTER9 |  | 53 | -0.35 | -1.44 | 0.035 | 0.270 | 1.000 | 1085 | tags=21%, list=8%, signal=23% |
| 68 | AGED\_MOUSE\_MUSCLE\_UP |  | 30 | -0.40 | -1.44 | 0.052 | 0.267 | 1.000 | 2390 | tags=33%, list=18%, signal=41% |
| 69 | CHR3P22 |  | 39 | -0.38 | -1.44 | 0.049 | 0.264 | 1.000 | 2489 | tags=41%, list=19%, signal=50% |
| 70 | GTATGAT,MIR-154,MIR-487 |  | 52 | -0.35 | -1.43 | 0.033 | 0.266 | 1.000 | 1547 | tags=17%, list=12%, signal=20% |
| 71 | ZHAN\_MM\_CD138\_LB\_VS\_REST |  | 24 | -0.42 | -1.43 | 0.074 | 0.266 | 1.000 | 2020 | tags=46%, list=15%, signal=54% |
| 72 | OVARIAN\_INFERTILITY\_GENES |  | 25 | -0.42 | -1.43 | 0.065 | 0.270 | 1.000 | 850 | tags=24%, list=6%, signal=26% |
| 73 | SHIPP\_FL\_VS\_DLBCL\_UP |  | 34 | -0.40 | -1.43 | 0.063 | 0.269 | 1.000 | 878 | tags=18%, list=7%, signal=19% |
| 74 | GTTGNYNNRGNAAC\_UNKNOWN |  | 65 | -0.34 | -1.42 | 0.034 | 0.266 | 1.000 | 2283 | tags=31%, list=17%, signal=37% |
| 75 | INSULIN\_ADIP\_SENS\_DN |  | 17 | -0.46 | -1.42 | 0.056 | 0.270 | 1.000 | 3398 | tags=53%, list=26%, signal=71% |
| 76 | TCANNTGAY\_V$SREBP1\_01 |  | 320 | -0.26 | -1.41 | 0.000 | 0.279 | 1.000 | 2289 | tags=26%, list=17%, signal=31% |
| 77 | PASSERINI\_EM |  | 34 | -0.38 | -1.41 | 0.059 | 0.283 | 1.000 | 3165 | tags=47%, list=24%, signal=62% |
| 78 | VEGF\_MMMEC\_6HRS\_UP |  | 49 | -0.35 | -1.41 | 0.026 | 0.280 | 1.000 | 3058 | tags=47%, list=23%, signal=61% |
| 79 | AGED\_MOUSE\_HYPOTH\_DN |  | 37 | -0.37 | -1.40 | 0.056 | 0.287 | 1.000 | 1813 | tags=24%, list=14%, signal=28% |
| 80 | CHR3P14 |  | 27 | -0.40 | -1.38 | 0.082 | 0.327 | 1.000 | 1302 | tags=30%, list=10%, signal=33% |
| 81 | FLECHNER\_KIDNEY\_TRANSPLANT\_REJECTION\_DN |  | 539 | -0.24 | -1.37 | 0.000 | 0.348 | 1.000 | 2355 | tags=24%, list=18%, signal=29% |
| 82 | BREAST\_CANCER\_ESTROGEN\_SIGNALING |  | 93 | -0.30 | -1.36 | 0.026 | 0.354 | 1.000 | 997 | tags=22%, list=7%, signal=23% |
| 83 | TGFBETA\_EARLY\_UP |  | 47 | -0.35 | -1.36 | 0.059 | 0.355 | 1.000 | 2124 | tags=36%, list=16%, signal=43% |
| 84 | CHR5Q33 |  | 30 | -0.38 | -1.36 | 0.080 | 0.364 | 1.000 | 3297 | tags=43%, list=25%, signal=57% |
| 85 | AGUIRRE\_PANCREAS\_CHR8 |  | 61 | -0.33 | -1.36 | 0.054 | 0.361 | 1.000 | 3309 | tags=41%, list=25%, signal=54% |
| 86 | HDACI\_COLON\_SUL12HRS\_DN |  | 22 | -0.41 | -1.36 | 0.079 | 0.358 | 1.000 | 1280 | tags=27%, list=10%, signal=30% |
| 87 | CHR3P26 |  | 15 | -0.44 | -1.35 | 0.097 | 0.366 | 1.000 | 1049 | tags=27%, list=8%, signal=29% |
| 88 | CHR5Q23 |  | 38 | -0.35 | -1.35 | 0.087 | 0.367 | 1.000 | 1694 | tags=29%, list=13%, signal=33% |
| 89 | V$AR\_03 |  | 33 | -0.37 | -1.35 | 0.078 | 0.368 | 1.000 | 861 | tags=21%, list=6%, signal=23% |
| 90 | LIZUKA\_L1\_GR\_G1 |  | 20 | -0.43 | -1.34 | 0.125 | 0.369 | 1.000 | 1563 | tags=35%, list=12%, signal=40% |
| 91 | LI\_FETAL\_VS\_WT\_KIDNEY\_UP |  | 179 | -0.27 | -1.34 | 0.018 | 0.370 | 1.000 | 1967 | tags=28%, list=15%, signal=33% |
| 92 | CHR17Q25 |  | 108 | -0.29 | -1.34 | 0.033 | 0.370 | 1.000 | 3269 | tags=42%, list=25%, signal=55% |
| 93 | PENG\_GLUTAMINE\_UP |  | 227 | -0.26 | -1.34 | 0.012 | 0.372 | 1.000 | 1838 | tags=23%, list=14%, signal=26% |
| 94 | V$CP2\_02 |  | 162 | -0.27 | -1.34 | 0.012 | 0.371 | 1.000 | 1826 | tags=23%, list=14%, signal=26% |
| 95 | FALT\_BCLL\_IG\_MUTATED\_VS\_WT\_DN |  | 48 | -0.33 | -1.33 | 0.070 | 0.373 | 1.000 | 2411 | tags=33%, list=18%, signal=41% |
| 96 | BECKER\_TAMOXIFEN\_RESISTANT\_UP |  | 40 | -0.35 | -1.33 | 0.086 | 0.372 | 1.000 | 1104 | tags=20%, list=8%, signal=22% |
| 97 | OXIDATIVE\_PHOSPHORYLATION |  | 55 | -0.32 | -1.32 | 0.083 | 0.388 | 1.000 | 2410 | tags=35%, list=18%, signal=42% |
| 98 | STOSSI\_ER\_UP |  | 47 | -0.33 | -1.32 | 0.087 | 0.390 | 1.000 | 1612 | tags=30%, list=12%, signal=34% |
| 99 | CHR3Q13 |  | 48 | -0.33 | -1.31 | 0.086 | 0.404 | 1.000 | 2264 | tags=27%, list=17%, signal=33% |
| 100 | NGUYEN\_KERATO\_UP |  | 27 | -0.37 | -1.31 | 0.104 | 0.403 | 1.000 | 1932 | tags=33%, list=15%, signal=39% |
| 101 | BAF57\_BT549\_UP |  | 185 | -0.26 | -1.31 | 0.018 | 0.408 | 1.000 | 1681 | tags=23%, list=13%, signal=26% |
| 102 | TSA\_HEPATOMA\_UP |  | 33 | -0.35 | -1.31 | 0.120 | 0.412 | 1.000 | 3219 | tags=42%, list=24%, signal=56% |
| 103 | HDACI\_COLON\_BUT24HRS\_UP |  | 61 | -0.31 | -1.30 | 0.068 | 0.412 | 1.000 | 3023 | tags=41%, list=23%, signal=53% |
| 104 | V$HEB\_Q6 |  | 178 | -0.26 | -1.30 | 0.009 | 0.419 | 1.000 | 2102 | tags=25%, list=16%, signal=30% |
| 105 | CHR2Q37 |  | 65 | -0.30 | -1.29 | 0.080 | 0.431 | 1.000 | 1948 | tags=25%, list=15%, signal=29% |
| 106 | BLOOD\_CLOTTING\_CASCADE |  | 20 | -0.41 | -1.29 | 0.154 | 0.428 | 1.000 | 195 | tags=10%, list=1%, signal=10% |
| 107 | NAKAJIMA\_MCSMBP\_MAST |  | 45 | -0.33 | -1.29 | 0.111 | 0.435 | 1.000 | 2746 | tags=31%, list=21%, signal=39% |
| 108 | ST\_WNT\_BETA\_CATENIN\_PATHWAY |  | 28 | -0.37 | -1.29 | 0.127 | 0.434 | 1.000 | 2345 | tags=32%, list=18%, signal=39% |
| 109 | HCC\_SURVIVAL\_GOOD\_VS\_POOR\_UP |  | 124 | -0.27 | -1.29 | 0.040 | 0.431 | 1.000 | 2019 | tags=19%, list=15%, signal=23% |
| 110 | ROSS\_MLL\_FUSION |  | 83 | -0.29 | -1.29 | 0.049 | 0.432 | 1.000 | 1219 | tags=22%, list=9%, signal=24% |
| 111 | BLEO\_HUMAN\_LYMPH\_HIGH\_4HRS\_UP |  | 21 | -0.39 | -1.28 | 0.157 | 0.434 | 1.000 | 3004 | tags=48%, list=23%, signal=61% |
| 112 | TGFBETA\_ALL\_UP |  | 80 | -0.28 | -1.28 | 0.083 | 0.444 | 1.000 | 2124 | tags=30%, list=16%, signal=35% |
| 113 | V$TATA\_C |  | 218 | -0.24 | -1.27 | 0.019 | 0.466 | 1.000 | 859 | tags=12%, list=6%, signal=13% |
| 114 | TGTYNNNNNRGCARM\_UNKNOWN |  | 52 | -0.31 | -1.27 | 0.107 | 0.462 | 1.000 | 1871 | tags=27%, list=14%, signal=31% |
| 115 | V$MIF1\_01 |  | 110 | -0.27 | -1.26 | 0.089 | 0.476 | 1.000 | 2163 | tags=25%, list=16%, signal=29% |
| 116 | BRG1\_H1299\_UP |  | 31 | -0.35 | -1.26 | 0.149 | 0.483 | 1.000 | 2359 | tags=32%, list=18%, signal=39% |
| 117 | CHR5Q21 |  | 17 | -0.41 | -1.25 | 0.185 | 0.502 | 1.000 | 3179 | tags=41%, list=24%, signal=54% |
| 118 | HDACI\_COLON\_BUT\_UP |  | 154 | -0.25 | -1.25 | 0.055 | 0.499 | 1.000 | 3023 | tags=33%, list=23%, signal=42% |
| 119 | H2O2\_CSBRESCUED\_C1\_UP |  | 40 | -0.33 | -1.25 | 0.134 | 0.501 | 1.000 | 682 | tags=20%, list=5%, signal=21% |
| 120 | H2O2\_CSBRESCUED\_UP |  | 54 | -0.30 | -1.25 | 0.119 | 0.500 | 1.000 | 682 | tags=19%, list=5%, signal=19% |
| 121 | V$SREBP1\_01 |  | 125 | -0.26 | -1.25 | 0.070 | 0.500 | 1.000 | 2187 | tags=30%, list=16%, signal=35% |
| 122 | TNFALPHA\_TGZ\_ADIP\_DN |  | 25 | -0.35 | -1.24 | 0.144 | 0.511 | 1.000 | 2196 | tags=32%, list=16%, signal=38% |
| 123 | MMS\_HUMAN\_LYMPH\_HIGH\_24HRS\_UP |  | 19 | -0.38 | -1.24 | 0.187 | 0.518 | 1.000 | 1039 | tags=21%, list=8%, signal=23% |
| 124 | KENNY\_WNT\_DN |  | 45 | -0.30 | -1.23 | 0.150 | 0.536 | 1.000 | 889 | tags=18%, list=7%, signal=19% |
| 125 | CHR5Q14 |  | 22 | -0.37 | -1.23 | 0.177 | 0.536 | 1.000 | 2700 | tags=45%, list=20%, signal=57% |
| 126 | FETAL\_LIVER\_VS\_ADULT\_LIVER\_GNF2 |  | 69 | -0.28 | -1.22 | 0.101 | 0.541 | 1.000 | 2086 | tags=25%, list=16%, signal=29% |
| 127 | GILDEA\_BLADDER\_UP |  | 30 | -0.34 | -1.22 | 0.157 | 0.537 | 1.000 | 1493 | tags=30%, list=11%, signal=34% |
| 128 | ACAACTT,MIR-382 |  | 57 | -0.29 | -1.22 | 0.110 | 0.533 | 1.000 | 1988 | tags=25%, list=15%, signal=29% |
| 129 | V$FOXO3\_01 |  | 171 | -0.24 | -1.22 | 0.062 | 0.552 | 1.000 | 1416 | tags=18%, list=11%, signal=19% |
| 130 | BUT\_TSA\_UP |  | 18 | -0.39 | -1.22 | 0.202 | 0.552 | 1.000 | 710 | tags=28%, list=5%, signal=29% |
| 131 | NDKDYNAMINPATHWAY |  | 17 | -0.41 | -1.21 | 0.189 | 0.551 | 1.000 | 226 | tags=12%, list=2%, signal=12% |
| 132 | VEGF\_HUVEC\_30MIN\_UP |  | 24 | -0.36 | -1.21 | 0.181 | 0.550 | 1.000 | 1694 | tags=25%, list=13%, signal=29% |
| 133 | CHESLER\_BRAIN\_CIS\_GENES |  | 50 | -0.30 | -1.21 | 0.163 | 0.553 | 1.000 | 1313 | tags=20%, list=10%, signal=22% |
| 134 | RNA\_TRANSCRIPTION\_REACTOME |  | 34 | -0.34 | -1.21 | 0.190 | 0.553 | 1.000 | 1008 | tags=24%, list=8%, signal=25% |
| 135 | HDACI\_COLON\_TSA\_DN |  | 55 | -0.29 | -1.20 | 0.160 | 0.568 | 1.000 | 1817 | tags=27%, list=14%, signal=31% |
| 136 | CHANG\_SERUM\_RESPONSE\_DN |  | 112 | -0.26 | -1.20 | 0.118 | 0.570 | 1.000 | 847 | tags=16%, list=6%, signal=17% |
| 137 | TNFALPHA\_ADIP\_DN |  | 54 | -0.29 | -1.20 | 0.169 | 0.569 | 1.000 | 1132 | tags=20%, list=8%, signal=22% |
| 138 | TPA\_SKIN\_DN |  | 15 | -0.41 | -1.20 | 0.241 | 0.574 | 1.000 | 640 | tags=20%, list=5%, signal=21% |
| 139 | HDACI\_COLON\_CUR12HRS\_UP |  | 16 | -0.39 | -1.20 | 0.211 | 0.572 | 1.000 | 1086 | tags=25%, list=8%, signal=27% |
| 140 | V$P300\_01 |  | 174 | -0.24 | -1.19 | 0.072 | 0.588 | 1.000 | 1580 | tags=18%, list=12%, signal=20% |
| 141 | GLUTATHIONE\_METABOLISM |  | 28 | -0.35 | -1.19 | 0.178 | 0.586 | 1.000 | 2665 | tags=43%, list=20%, signal=53% |
| 142 | IGF\_VS\_PDGF\_DN |  | 34 | -0.32 | -1.19 | 0.171 | 0.583 | 1.000 | 2642 | tags=35%, list=20%, signal=44% |
| 143 | V$USF\_Q6 |  | 183 | -0.24 | -1.19 | 0.085 | 0.583 | 1.000 | 2531 | tags=30%, list=19%, signal=37% |
| 144 | CMV\_ALL\_DN |  | 99 | -0.26 | -1.19 | 0.123 | 0.591 | 1.000 | 1628 | tags=22%, list=12%, signal=25% |
| 145 | 4NQO\_UNIQUE\_FIBRO\_UP |  | 22 | -0.36 | -1.18 | 0.225 | 0.593 | 1.000 | 1396 | tags=23%, list=10%, signal=25% |
| 146 | SERUM\_FIBROBLAST\_CORE\_DN |  | 160 | -0.24 | -1.18 | 0.105 | 0.597 | 1.000 | 2634 | tags=31%, list=20%, signal=38% |
| 147 | ADIP\_VS\_PREADIP\_UP |  | 35 | -0.31 | -1.17 | 0.207 | 0.618 | 1.000 | 2980 | tags=46%, list=22%, signal=59% |
| 148 | YTAAYNGCT\_UNKNOWN |  | 108 | -0.25 | -1.17 | 0.136 | 0.623 | 1.000 | 2213 | tags=24%, list=17%, signal=29% |
| 149 | GLUTAMATE\_METABOLISM |  | 23 | -0.35 | -1.17 | 0.241 | 0.621 | 1.000 | 1498 | tags=30%, list=11%, signal=34% |
| 150 | V$LBP1\_Q6 |  | 147 | -0.24 | -1.17 | 0.118 | 0.622 | 1.000 | 1127 | tags=14%, list=8%, signal=15% |
| 151 | SCGGAAGY\_V$ELK1\_02 |  | 764 | -0.20 | -1.17 | 0.013 | 0.621 | 1.000 | 2274 | tags=23%, list=17%, signal=26% |
| 152 | GTTRYCATRR\_UNKNOWN |  | 105 | -0.25 | -1.17 | 0.164 | 0.625 | 1.000 | 1553 | tags=17%, list=12%, signal=19% |
| 153 | IFNALPHA\_RESIST\_DN |  | 18 | -0.37 | -1.17 | 0.223 | 0.621 | 1.000 | 1321 | tags=28%, list=10%, signal=31% |
| 154 | GO\_ROS |  | 25 | -0.34 | -1.16 | 0.226 | 0.637 | 1.000 | 1498 | tags=32%, list=11%, signal=36% |
| 155 | CMV\_24HRS\_DN |  | 70 | -0.27 | -1.16 | 0.168 | 0.634 | 1.000 | 1493 | tags=23%, list=11%, signal=26% |
| 156 | BRCA1\_OVEREXP\_UP |  | 157 | -0.24 | -1.16 | 0.135 | 0.631 | 1.000 | 1896 | tags=25%, list=14%, signal=29% |
| 157 | CHR4Q22 |  | 21 | -0.35 | -1.16 | 0.252 | 0.629 | 1.000 | 2738 | tags=38%, list=21%, signal=48% |
| 158 | V$SRY\_01 |  | 144 | -0.24 | -1.16 | 0.146 | 0.627 | 1.000 | 1311 | tags=16%, list=10%, signal=18% |
| 159 | ADIP\_VS\_FIBRO\_UP |  | 34 | -0.31 | -1.16 | 0.216 | 0.627 | 1.000 | 1868 | tags=35%, list=14%, signal=41% |
| 160 | DORSAM\_HOXA9\_DN |  | 30 | -0.32 | -1.16 | 0.239 | 0.632 | 1.000 | 1818 | tags=20%, list=14%, signal=23% |
| 161 | HDACI\_COLON\_TSA48HRS\_UP |  | 35 | -0.30 | -1.15 | 0.234 | 0.634 | 1.000 | 2388 | tags=34%, list=18%, signal=42% |
| 162 | UREA\_CYCLE\_AND\_METABOLISM\_OF\_AMINO\_GROUPS |  | 17 | -0.37 | -1.15 | 0.253 | 0.630 | 1.000 | 873 | tags=24%, list=7%, signal=25% |
| 163 | V$RFX1\_02 |  | 168 | -0.23 | -1.15 | 0.123 | 0.635 | 1.000 | 1897 | tags=19%, list=14%, signal=22% |
| 164 | ROSS\_CBF |  | 82 | -0.26 | -1.15 | 0.190 | 0.633 | 1.000 | 2753 | tags=34%, list=21%, signal=43% |
| 165 | VIPPATHWAY |  | 25 | -0.34 | -1.15 | 0.241 | 0.634 | 1.000 | 2343 | tags=20%, list=18%, signal=24% |
| 166 | CHR14Q31 |  | 15 | -0.39 | -1.15 | 0.262 | 0.633 | 1.000 | 3031 | tags=40%, list=23%, signal=52% |
| 167 | ET743PT650\_COLONCA\_DN |  | 44 | -0.30 | -1.14 | 0.229 | 0.644 | 1.000 | 2080 | tags=27%, list=16%, signal=32% |
| 168 | UVC\_TTD\_4HR\_UP |  | 58 | -0.28 | -1.14 | 0.222 | 0.641 | 1.000 | 2124 | tags=24%, list=16%, signal=29% |
| 169 | ADIP\_DIFF\_UP |  | 64 | -0.27 | -1.14 | 0.182 | 0.653 | 1.000 | 1132 | tags=20%, list=8%, signal=22% |
| 170 | WCAANNNYCAG\_UNKNOWN |  | 157 | -0.23 | -1.14 | 0.152 | 0.655 | 1.000 | 1340 | tags=15%, list=10%, signal=17% |
| 171 | NAB\_LUNG\_DN |  | 53 | -0.28 | -1.13 | 0.230 | 0.673 | 1.000 | 1227 | tags=19%, list=9%, signal=21% |
| 172 | BRG1\_ALAB\_DN |  | 32 | -0.31 | -1.13 | 0.242 | 0.681 | 1.000 | 1205 | tags=28%, list=9%, signal=31% |
| 173 | CHR1Q41 |  | 29 | -0.32 | -1.13 | 0.249 | 0.681 | 1.000 | 2484 | tags=31%, list=19%, signal=38% |
| 174 | V$EGR1\_01 |  | 181 | -0.22 | -1.13 | 0.166 | 0.682 | 1.000 | 1694 | tags=18%, list=13%, signal=21% |
| 175 | CHR14Q13 |  | 22 | -0.34 | -1.13 | 0.275 | 0.682 | 1.000 | 2699 | tags=27%, list=20%, signal=34% |
| 176 | ATCTTGC,MIR-31 |  | 48 | -0.28 | -1.13 | 0.235 | 0.679 | 1.000 | 833 | tags=15%, list=6%, signal=16% |
| 177 | PORPHYRIN\_AND\_CHLOROPHYLL\_METABOLISM |  | 20 | -0.34 | -1.12 | 0.270 | 0.678 | 1.000 | 1166 | tags=20%, list=9%, signal=22% |
| 178 | DIAB\_NEPH\_UP |  | 60 | -0.27 | -1.12 | 0.243 | 0.680 | 1.000 | 2301 | tags=28%, list=17%, signal=34% |
| 179 | CMV\_HCMV\_TIMECOURSE\_6HRS\_DN |  | 52 | -0.28 | -1.12 | 0.248 | 0.677 | 1.000 | 1385 | tags=19%, list=10%, signal=21% |
| 180 | V$GR\_01 |  | 136 | -0.23 | -1.12 | 0.200 | 0.677 | 1.000 | 880 | tags=13%, list=7%, signal=13% |
| 181 | CHR20Q12 |  | 32 | -0.31 | -1.12 | 0.262 | 0.675 | 1.000 | 797 | tags=16%, list=6%, signal=17% |
| 182 | AAGWWRNYGGC\_UNKNOWN |  | 86 | -0.25 | -1.12 | 0.235 | 0.679 | 1.000 | 2321 | tags=24%, list=17%, signal=29% |
| 183 | POD1\_KO\_DN |  | 592 | -0.20 | -1.12 | 0.070 | 0.682 | 1.000 | 2799 | tags=28%, list=21%, signal=33% |
| 184 | CHR17Q11 |  | 74 | -0.26 | -1.12 | 0.257 | 0.681 | 1.000 | 2755 | tags=30%, list=21%, signal=37% |
| 185 | ABBUD\_LIF\_UP |  | 38 | -0.29 | -1.12 | 0.270 | 0.684 | 1.000 | 518 | tags=13%, list=4%, signal=14% |
| 186 | UVC\_LOW\_ALL\_UP |  | 19 | -0.35 | -1.11 | 0.310 | 0.684 | 1.000 | 710 | tags=26%, list=5%, signal=28% |
| 187 | ROS\_MOUSE\_AORTA\_UP |  | 22 | -0.34 | -1.11 | 0.301 | 0.689 | 1.000 | 1300 | tags=27%, list=10%, signal=30% |
| 188 | HEMATOP\_STEM\_ALL\_UP |  | 35 | -0.30 | -1.11 | 0.307 | 0.698 | 1.000 | 1104 | tags=20%, list=8%, signal=22% |
| 189 | ROSS\_AML1\_ETO |  | 82 | -0.25 | -1.11 | 0.239 | 0.695 | 1.000 | 2753 | tags=37%, list=21%, signal=46% |
| 190 | CHR12Q24 |  | 148 | -0.22 | -1.11 | 0.230 | 0.695 | 1.000 | 2659 | tags=24%, list=20%, signal=29% |
| 191 | V$HSF2\_01 |  | 164 | -0.22 | -1.11 | 0.186 | 0.693 | 1.000 | 2018 | tags=18%, list=15%, signal=21% |
| 192 | AAAYWAACM\_V$HFH4\_01 |  | 180 | -0.22 | -1.11 | 0.192 | 0.690 | 1.000 | 1453 | tags=15%, list=11%, signal=17% |
| 193 | CHR15Q15 |  | 41 | -0.29 | -1.10 | 0.259 | 0.698 | 1.000 | 2355 | tags=29%, list=18%, signal=35% |
| 194 | YGACNNYACAR\_UNKNOWN |  | 59 | -0.26 | -1.10 | 0.274 | 0.698 | 1.000 | 1484 | tags=12%, list=11%, signal=13% |
| 195 | CPR\_LOW\_LIVER\_DN |  | 21 | -0.34 | -1.10 | 0.301 | 0.696 | 1.000 | 1694 | tags=33%, list=13%, signal=38% |
| 196 | STRESS\_GENOTOXIC\_SPECIFIC\_UP |  | 33 | -0.30 | -1.10 | 0.275 | 0.710 | 1.000 | 2737 | tags=36%, list=21%, signal=46% |
| 197 | V$AREB6\_03 |  | 164 | -0.22 | -1.10 | 0.217 | 0.707 | 1.000 | 1851 | tags=20%, list=14%, signal=22% |
| 198 | POMEROY\_DESMOPLASIC\_VS\_CLASSIC\_MD\_DN |  | 41 | -0.28 | -1.10 | 0.296 | 0.706 | 1.000 | 883 | tags=12%, list=7%, signal=13% |
| 199 | IGF1RPATHWAY |  | 15 | -0.36 | -1.09 | 0.326 | 0.709 | 1.000 | 2345 | tags=33%, list=18%, signal=40% |
| 200 | GH\_AUTOCRINE\_UP |  | 180 | -0.21 | -1.09 | 0.194 | 0.726 | 1.000 | 1277 | tags=15%, list=10%, signal=16% |
| 201 | CMV\_HCMV\_TIMECOURSE\_16HRS\_DN |  | 20 | -0.35 | -1.09 | 0.344 | 0.725 | 1.000 | 1335 | tags=25%, list=10%, signal=28% |
| 202 | CIS\_XPC\_DN |  | 182 | -0.22 | -1.09 | 0.219 | 0.723 | 1.000 | 2591 | tags=27%, list=19%, signal=33% |
| 203 | GOLUB\_ALL\_VS\_AML\_DN |  | 16 | -0.36 | -1.09 | 0.355 | 0.723 | 1.000 | 1239 | tags=31%, list=9%, signal=34% |
| 204 | HSIAO\_LIVER\_SPECIFIC\_GENES |  | 231 | -0.21 | -1.08 | 0.228 | 0.732 | 1.000 | 2019 | tags=16%, list=15%, signal=19% |
| 205 | FATTY\_ACID\_METABOLISM |  | 79 | -0.24 | -1.08 | 0.299 | 0.733 | 1.000 | 3345 | tags=35%, list=25%, signal=47% |
| 206 | FSH\_OVARY\_MCV152\_UP |  | 61 | -0.26 | -1.08 | 0.278 | 0.733 | 1.000 | 1952 | tags=26%, list=15%, signal=31% |
| 207 | V$MYB\_Q6 |  | 171 | -0.22 | -1.08 | 0.245 | 0.731 | 1.000 | 1834 | tags=19%, list=14%, signal=22% |
| 208 | CGTSACG\_V$PAX3\_B |  | 99 | -0.23 | -1.08 | 0.295 | 0.728 | 1.000 | 2194 | tags=24%, list=16%, signal=29% |
| 209 | CHR1P13 |  | 75 | -0.25 | -1.08 | 0.303 | 0.727 | 1.000 | 1180 | tags=15%, list=9%, signal=16% |
| 210 | IDX\_TSA\_DN\_CLUSTER5 |  | 42 | -0.28 | -1.08 | 0.329 | 0.725 | 1.000 | 1712 | tags=26%, list=13%, signal=30% |
| 211 | CARIES\_PULP\_DN |  | 70 | -0.25 | -1.08 | 0.295 | 0.723 | 1.000 | 2751 | tags=34%, list=21%, signal=43% |
| 212 | V$SRF\_C |  | 163 | -0.21 | -1.08 | 0.264 | 0.736 | 1.000 | 1874 | tags=21%, list=14%, signal=24% |
| 213 | FALT\_BCLL\_DN |  | 49 | -0.26 | -1.07 | 0.314 | 0.738 | 1.000 | 1279 | tags=18%, list=10%, signal=20% |
| 214 | TGGNNNNNNKCCAR\_UNKNOWN |  | 284 | -0.20 | -1.07 | 0.218 | 0.736 | 1.000 | 2123 | tags=22%, list=16%, signal=25% |
| 215 | GGGNRMNNYCAT\_UNKNOWN |  | 57 | -0.26 | -1.07 | 0.316 | 0.733 | 1.000 | 3243 | tags=39%, list=24%, signal=51% |
| 216 | HDACI\_COLON\_CURSUL\_UP |  | 37 | -0.28 | -1.07 | 0.324 | 0.731 | 1.000 | 1086 | tags=16%, list=8%, signal=18% |
| 217 | GTCNYYATGR\_UNKNOWN |  | 75 | -0.24 | -1.07 | 0.302 | 0.728 | 1.000 | 2437 | tags=31%, list=18%, signal=37% |
| 218 | CHR14Q32 |  | 94 | -0.24 | -1.07 | 0.295 | 0.728 | 1.000 | 2523 | tags=27%, list=19%, signal=33% |
| 219 | SIG\_CD40PATHWAYMAP |  | 33 | -0.29 | -1.07 | 0.337 | 0.728 | 1.000 | 1484 | tags=21%, list=11%, signal=24% |
| 220 | CHR7P21 |  | 24 | -0.32 | -1.07 | 0.350 | 0.732 | 1.000 | 460 | tags=17%, list=3%, signal=17% |
| 221 | NAKAJIMA\_MCSMBP\_EOS |  | 27 | -0.30 | -1.07 | 0.358 | 0.729 | 1.000 | 1569 | tags=26%, list=12%, signal=29% |
| 222 | V$FAC1\_01 |  | 149 | -0.21 | -1.07 | 0.285 | 0.738 | 1.000 | 1222 | tags=15%, list=9%, signal=16% |
| 223 | V$HMX1\_01 |  | 34 | -0.29 | -1.07 | 0.353 | 0.740 | 1.000 | 2293 | tags=26%, list=17%, signal=32% |
| 224 | CERAMIDEPATHWAY |  | 22 | -0.31 | -1.06 | 0.336 | 0.739 | 1.000 | 2343 | tags=32%, list=18%, signal=39% |
| 225 | V$GABP\_B |  | 155 | -0.22 | -1.06 | 0.293 | 0.736 | 1.000 | 2379 | tags=23%, list=18%, signal=28% |
| 226 | OLDONLY\_FIBRO\_DN |  | 46 | -0.27 | -1.06 | 0.328 | 0.735 | 1.000 | 1370 | tags=20%, list=10%, signal=22% |
| 227 | V$USF\_02 |  | 194 | -0.21 | -1.06 | 0.279 | 0.732 | 1.000 | 1222 | tags=16%, list=9%, signal=18% |
| 228 | AS3\_FIBRO\_C2 |  | 31 | -0.29 | -1.06 | 0.370 | 0.733 | 1.000 | 1017 | tags=19%, list=8%, signal=21% |
| 229 | OXSTRESS\_RPE\_H2O2TBH\_DN |  | 31 | -0.29 | -1.06 | 0.378 | 0.739 | 1.000 | 1017 | tags=19%, list=8%, signal=21% |
| 230 | V$TFIII\_Q6 |  | 145 | -0.21 | -1.06 | 0.293 | 0.742 | 1.000 | 2846 | tags=27%, list=21%, signal=34% |
| 231 | CHR10Q23 |  | 62 | -0.25 | -1.06 | 0.369 | 0.740 | 1.000 | 2445 | tags=27%, list=18%, signal=33% |
| 232 | V$TEF\_Q6 |  | 177 | -0.21 | -1.06 | 0.309 | 0.738 | 1.000 | 1076 | tags=14%, list=8%, signal=15% |
| 233 | TTTGCAC,MIR-19A,MIR-19B |  | 393 | -0.19 | -1.06 | 0.227 | 0.742 | 1.000 | 2234 | tags=21%, list=17%, signal=24% |
| 234 | CROONQUIST\_RAS\_STROMA\_UP |  | 23 | -0.32 | -1.05 | 0.376 | 0.745 | 1.000 | 677 | tags=17%, list=5%, signal=18% |
| 235 | GTGACGY\_V$E4F1\_Q6 |  | 442 | -0.19 | -1.05 | 0.235 | 0.743 | 1.000 | 2194 | tags=21%, list=16%, signal=24% |
| 236 | IDX\_TSA\_DN\_CLUSTER3 |  | 69 | -0.24 | -1.05 | 0.316 | 0.743 | 1.000 | 2810 | tags=36%, list=21%, signal=46% |
| 237 | NELSON\_ANDROGEN\_UP |  | 59 | -0.25 | -1.05 | 0.353 | 0.740 | 1.000 | 622 | tags=12%, list=5%, signal=12% |
| 238 | HDACI\_COLON\_TSA12HRS\_UP |  | 20 | -0.32 | -1.05 | 0.361 | 0.740 | 1.000 | 1113 | tags=25%, list=8%, signal=27% |
| 239 | CHR1P21 |  | 19 | -0.34 | -1.05 | 0.377 | 0.741 | 1.000 | 1182 | tags=21%, list=9%, signal=23% |
| 240 | CHR1Q32 |  | 101 | -0.23 | -1.05 | 0.342 | 0.740 | 1.000 | 1554 | tags=18%, list=12%, signal=20% |
| 241 | AKTPATHWAY |  | 17 | -0.35 | -1.05 | 0.354 | 0.739 | 1.000 | 3062 | tags=29%, list=23%, signal=38% |
| 242 | PENG\_RAPAMYCIN\_UP |  | 154 | -0.21 | -1.05 | 0.329 | 0.738 | 1.000 | 2649 | tags=27%, list=20%, signal=34% |
| 243 | V$AP2\_Q6\_01 |  | 180 | -0.21 | -1.05 | 0.319 | 0.739 | 1.000 | 2556 | tags=25%, list=19%, signal=31% |
| 244 | V$SMAD\_Q6 |  | 175 | -0.21 | -1.05 | 0.285 | 0.740 | 1.000 | 1002 | tags=12%, list=8%, signal=13% |
| 245 | CHR19P13 |  | 343 | -0.19 | -1.05 | 0.273 | 0.740 | 1.000 | 1910 | tags=16%, list=14%, signal=18% |
| 246 | AGEING\_KIDNEY\_SPECIFIC\_DN |  | 120 | -0.22 | -1.05 | 0.321 | 0.742 | 1.000 | 2262 | tags=29%, list=17%, signal=35% |
| 247 | NOUZOVA\_CPG\_H4\_UP |  | 95 | -0.23 | -1.04 | 0.354 | 0.752 | 1.000 | 2671 | tags=28%, list=20%, signal=35% |
| 248 | AS3\_FIBRO\_C1 |  | 31 | -0.29 | -1.04 | 0.386 | 0.750 | 1.000 | 1017 | tags=19%, list=8%, signal=21% |
| 249 | EGF\_HDMEC\_UP |  | 42 | -0.27 | -1.04 | 0.385 | 0.747 | 1.000 | 984 | tags=17%, list=7%, signal=18% |
| 250 | GTACTGT,MIR-101 |  | 200 | -0.21 | -1.04 | 0.295 | 0.747 | 1.000 | 2194 | tags=25%, list=16%, signal=29% |
| 251 | V$MAZR\_01 |  | 158 | -0.21 | -1.04 | 0.368 | 0.745 | 1.000 | 1270 | tags=15%, list=10%, signal=17% |
| 252 | CHR15Q22 |  | 54 | -0.26 | -1.04 | 0.384 | 0.743 | 1.000 | 1424 | tags=19%, list=11%, signal=21% |
| 253 | V$PAX3\_B |  | 66 | -0.24 | -1.04 | 0.382 | 0.742 | 1.000 | 859 | tags=15%, list=6%, signal=16% |
| 254 | CHAUVIN\_ANDROGEN\_REGULATED\_GENES |  | 43 | -0.27 | -1.04 | 0.388 | 0.742 | 1.000 | 1448 | tags=23%, list=11%, signal=26% |
| 255 | CHR11Q12 |  | 84 | -0.24 | -1.04 | 0.387 | 0.740 | 1.000 | 1478 | tags=14%, list=11%, signal=16% |
| 256 | CHR9Q32 |  | 19 | -0.32 | -1.04 | 0.410 | 0.743 | 1.000 | 1544 | tags=21%, list=12%, signal=24% |
| 257 | NING\_COPD\_UP |  | 142 | -0.21 | -1.04 | 0.367 | 0.741 | 1.000 | 1806 | tags=20%, list=14%, signal=23% |
| 258 | INSULIN\_NIH3T3\_UP |  | 16 | -0.34 | -1.04 | 0.437 | 0.744 | 1.000 | 1114 | tags=25%, list=8%, signal=27% |
| 259 | CHR15Q21 |  | 61 | -0.25 | -1.03 | 0.380 | 0.745 | 1.000 | 1558 | tags=21%, list=12%, signal=24% |
| 260 | V$NRF2\_01 |  | 167 | -0.21 | -1.03 | 0.325 | 0.745 | 1.000 | 2267 | tags=23%, list=17%, signal=28% |
| 261 | V$PR\_Q2 |  | 165 | -0.21 | -1.03 | 0.363 | 0.743 | 1.000 | 1391 | tags=13%, list=10%, signal=15% |
| 262 | BCL2\_FAMILY\_AND\_REG\_NETWORK |  | 21 | -0.31 | -1.03 | 0.408 | 0.754 | 1.000 | 890 | tags=19%, list=7%, signal=20% |
| 263 | YTCCCRNNAGGY\_UNKNOWN |  | 46 | -0.26 | -1.03 | 0.425 | 0.752 | 1.000 | 1663 | tags=22%, list=12%, signal=25% |
| 264 | IGLESIAS\_E2FMINUS\_UP |  | 136 | -0.21 | -1.03 | 0.368 | 0.754 | 1.000 | 1871 | tags=21%, list=14%, signal=25% |
| 265 | NING\_COPD\_DN |  | 111 | -0.22 | -1.03 | 0.369 | 0.753 | 1.000 | 1908 | tags=22%, list=14%, signal=25% |
| 266 | AGED\_MOUSE\_CORTEX\_DN |  | 44 | -0.26 | -1.03 | 0.384 | 0.752 | 1.000 | 1458 | tags=18%, list=11%, signal=20% |
| 267 | V$AHR\_Q5 |  | 147 | -0.21 | -1.03 | 0.401 | 0.759 | 1.000 | 2701 | tags=27%, list=20%, signal=34% |
| 268 | LE\_MYELIN\_DN |  | 87 | -0.23 | -1.02 | 0.384 | 0.763 | 1.000 | 1958 | tags=21%, list=15%, signal=24% |
| 269 | STRESSPATHWAY |  | 24 | -0.30 | -1.02 | 0.431 | 0.765 | 1.000 | 2343 | tags=29%, list=18%, signal=35% |
| 270 | ACTGCAG,MIR-17-3P |  | 79 | -0.23 | -1.02 | 0.402 | 0.767 | 1.000 | 1565 | tags=20%, list=12%, signal=23% |
| 271 | CHR10Q25 |  | 32 | -0.28 | -1.02 | 0.410 | 0.769 | 1.000 | 1303 | tags=22%, list=10%, signal=24% |
| 272 | CHR18Q21 |  | 53 | -0.25 | -1.02 | 0.404 | 0.767 | 1.000 | 2112 | tags=28%, list=16%, signal=34% |
| 273 | CHR15Q23 |  | 19 | -0.31 | -1.02 | 0.420 | 0.766 | 1.000 | 2129 | tags=32%, list=16%, signal=38% |
| 274 | ACTGTGA,MIR-27A,MIR-27B |  | 347 | -0.18 | -1.02 | 0.393 | 0.773 | 1.000 | 2166 | tags=20%, list=16%, signal=23% |
| 275 | V$SP1\_Q6\_01 |  | 156 | -0.21 | -1.01 | 0.427 | 0.777 | 1.000 | 1988 | tags=19%, list=15%, signal=22% |
| 276 | STARCH\_AND\_SUCROSE\_METABOLISM |  | 31 | -0.28 | -1.01 | 0.443 | 0.777 | 1.000 | 1366 | tags=19%, list=10%, signal=22% |
| 277 | GAMMA-UV\_FIBRO\_UP |  | 32 | -0.27 | -1.01 | 0.403 | 0.786 | 1.000 | 885 | tags=16%, list=7%, signal=17% |
| 278 | MITOCHONDRIAL\_FATTY\_ACID\_BETAOXIDATION |  | 16 | -0.33 | -1.01 | 0.427 | 0.794 | 1.000 | 508 | tags=19%, list=4%, signal=19% |
| 279 | WNTPATHWAY |  | 25 | -0.29 | -1.01 | 0.424 | 0.795 | 1.000 | 885 | tags=16%, list=7%, signal=17% |
| 280 | TAKEDA\_NUP8\_HOXA9\_6H\_DN |  | 32 | -0.27 | -1.01 | 0.434 | 0.793 | 1.000 | 2099 | tags=25%, list=16%, signal=30% |
| 281 | ST\_P38\_MAPK\_PATHWAY |  | 35 | -0.27 | -1.01 | 0.431 | 0.791 | 1.000 | 2345 | tags=29%, list=18%, signal=35% |
| 282 | RELAPATHWAY |  | 16 | -0.33 | -1.01 | 0.413 | 0.790 | 1.000 | 2350 | tags=38%, list=18%, signal=45% |
| 283 | CHR19Q13 |  | 457 | -0.18 | -1.00 | 0.459 | 0.791 | 1.000 | 1955 | tags=16%, list=15%, signal=18% |
| 284 | CHESLER\_BRAIN\_ONLY\_SUBSET |  | 23 | -0.30 | -1.00 | 0.461 | 0.790 | 1.000 | 162 | tags=13%, list=1%, signal=13% |
| 285 | RACTNNRTTTNC\_UNKNOWN |  | 84 | -0.22 | -1.00 | 0.424 | 0.791 | 1.000 | 2875 | tags=33%, list=22%, signal=42% |
| 286 | CROONQUIST\_RAS\_STROMA\_DN |  | 19 | -0.32 | -1.00 | 0.477 | 0.793 | 1.000 | 2924 | tags=53%, list=22%, signal=67% |
| 287 | HDACI\_COLON\_CUR\_DN |  | 38 | -0.27 | -1.00 | 0.449 | 0.793 | 1.000 | 1686 | tags=29%, list=13%, signal=33% |
| 288 | CHR14Q21 |  | 25 | -0.30 | -1.00 | 0.448 | 0.791 | 1.000 | 2963 | tags=32%, list=22%, signal=41% |
| 289 | V$HEN1\_01 |  | 134 | -0.20 | -1.00 | 0.484 | 0.790 | 1.000 | 2015 | tags=19%, list=15%, signal=23% |
| 290 | HYPOPHYSECTOMY\_RAT\_DN |  | 48 | -0.25 | -1.00 | 0.460 | 0.789 | 1.000 | 1924 | tags=25%, list=14%, signal=29% |
| 291 | V$ETS\_Q4 |  | 165 | -0.20 | -1.00 | 0.445 | 0.789 | 1.000 | 1167 | tags=13%, list=9%, signal=14% |
| 292 | V$PAX2\_02 |  | 181 | -0.20 | -1.00 | 0.447 | 0.788 | 1.000 | 1487 | tags=17%, list=11%, signal=18% |
| 293 | SCHRAETS\_MLL\_UP |  | 32 | -0.28 | -1.00 | 0.415 | 0.787 | 1.000 | 1645 | tags=22%, list=12%, signal=25% |
| 294 | IDX\_TSA\_UP\_CLUSTER6 |  | 146 | -0.20 | -1.00 | 0.455 | 0.785 | 1.000 | 1938 | tags=22%, list=15%, signal=25% |
| 295 | BCNU\_GLIOMA\_NOMGMT\_48HRS\_DN |  | 31 | -0.27 | -1.00 | 0.438 | 0.782 | 1.000 | 2406 | tags=29%, list=18%, signal=35% |
| 296 | CMV\_HCMV\_TIMECOURSE\_12HRS\_UP |  | 26 | -0.29 | -1.00 | 0.426 | 0.780 | 1.000 | 961 | tags=15%, list=7%, signal=17% |
| 297 | V$DR3\_Q4 |  | 97 | -0.22 | -1.00 | 0.447 | 0.781 | 1.000 | 823 | tags=12%, list=6%, signal=13% |
| 298 | CMV\_8HRS\_DN |  | 42 | -0.26 | -1.00 | 0.446 | 0.783 | 1.000 | 1193 | tags=19%, list=9%, signal=21% |
| 299 | CPR\_NULL\_LIVER\_UP |  | 32 | -0.28 | -0.99 | 0.448 | 0.785 | 1.000 | 572 | tags=13%, list=4%, signal=13% |
| 300 | HISTIDINE\_METABOLISM |  | 24 | -0.29 | -0.99 | 0.447 | 0.784 | 1.000 | 2570 | tags=38%, list=19%, signal=46% |
| 301 | V$STAT3\_02 |  | 105 | -0.21 | -0.99 | 0.493 | 0.796 | 1.000 | 1476 | tags=17%, list=11%, signal=19% |
| 302 | BRCA1\_SW480\_UP |  | 25 | -0.29 | -0.99 | 0.452 | 0.796 | 1.000 | 575 | tags=16%, list=4%, signal=17% |
| 303 | INOS\_ALL\_DN |  | 73 | -0.23 | -0.99 | 0.456 | 0.795 | 1.000 | 1124 | tags=15%, list=8%, signal=16% |
| 304 | CMV\_HCMV\_TIMECOURSE\_48HRS\_DN |  | 110 | -0.21 | -0.99 | 0.491 | 0.793 | 1.000 | 1722 | tags=17%, list=13%, signal=20% |
| 305 | V$S8\_01 |  | 172 | -0.20 | -0.99 | 0.435 | 0.791 | 1.000 | 1374 | tags=15%, list=10%, signal=17% |
| 306 | YAO\_P4\_KO\_VS\_WT\_UP |  | 65 | -0.23 | -0.99 | 0.476 | 0.790 | 1.000 | 2905 | tags=28%, list=22%, signal=35% |
| 307 | IRS1\_KO\_ADIP\_DN |  | 106 | -0.21 | -0.99 | 0.465 | 0.790 | 1.000 | 2106 | tags=21%, list=16%, signal=24% |
| 308 | CHR10Q22 |  | 57 | -0.24 | -0.98 | 0.479 | 0.801 | 1.000 | 2047 | tags=26%, list=15%, signal=31% |
| 309 | V$AP4\_01 |  | 176 | -0.20 | -0.98 | 0.513 | 0.800 | 1.000 | 2406 | tags=24%, list=18%, signal=29% |
| 310 | DORSEY\_DOXYCYCLINE\_UP |  | 29 | -0.28 | -0.98 | 0.447 | 0.799 | 1.000 | 1979 | tags=28%, list=15%, signal=32% |
| 311 | V$HEN1\_02 |  | 127 | -0.20 | -0.98 | 0.494 | 0.797 | 1.000 | 1964 | tags=18%, list=15%, signal=21% |
| 312 | GGCNRNWCTTYS\_UNKNOWN |  | 53 | -0.25 | -0.98 | 0.481 | 0.801 | 1.000 | 1984 | tags=28%, list=15%, signal=33% |
| 313 | ATGGYGGA\_UNKNOWN |  | 68 | -0.23 | -0.98 | 0.469 | 0.799 | 1.000 | 1391 | tags=16%, list=10%, signal=18% |
| 314 | DAVIES\_N |  | 19 | -0.32 | -0.98 | 0.505 | 0.799 | 1.000 | 574 | tags=16%, list=4%, signal=16% |
| 315 | CHR1P33 |  | 26 | -0.28 | -0.98 | 0.496 | 0.801 | 1.000 | 362 | tags=12%, list=3%, signal=12% |
| 316 | NAB\_LUNG\_UP |  | 26 | -0.29 | -0.98 | 0.478 | 0.799 | 1.000 | 2259 | tags=35%, list=17%, signal=42% |
| 317 | GGCNNMSMYNTTG\_UNKNOWN |  | 55 | -0.24 | -0.98 | 0.494 | 0.798 | 1.000 | 2564 | tags=29%, list=19%, signal=36% |
| 318 | AD12\_24HRS\_DN |  | 18 | -0.32 | -0.98 | 0.496 | 0.796 | 1.000 | 1818 | tags=28%, list=14%, signal=32% |
| 319 | V$CMYB\_01 |  | 170 | -0.19 | -0.97 | 0.518 | 0.804 | 1.000 | 2148 | tags=22%, list=16%, signal=26% |
| 320 | V$NGFIC\_01 |  | 177 | -0.19 | -0.97 | 0.557 | 0.810 | 1.000 | 1694 | tags=16%, list=13%, signal=18% |
| 321 | CMV\_HCMV\_TIMECOURSE\_24HRS\_DN |  | 42 | -0.25 | -0.97 | 0.546 | 0.814 | 1.000 | 2113 | tags=26%, list=16%, signal=31% |
| 322 | V$HTF\_01 |  | 47 | -0.24 | -0.97 | 0.504 | 0.817 | 1.000 | 2107 | tags=28%, list=16%, signal=33% |
| 323 | GH\_EXOGENOUS\_ALL\_UP |  | 46 | -0.24 | -0.97 | 0.530 | 0.816 | 1.000 | 1544 | tags=15%, list=12%, signal=17% |
| 324 | TAATAAT,MIR-126 |  | 162 | -0.19 | -0.97 | 0.563 | 0.815 | 1.000 | 1521 | tags=15%, list=11%, signal=17% |
| 325 | WTTGKCTG\_UNKNOWN |  | 347 | -0.18 | -0.97 | 0.623 | 0.818 | 1.000 | 1982 | tags=18%, list=15%, signal=20% |
| 326 | V$SRF\_01 |  | 42 | -0.25 | -0.97 | 0.508 | 0.817 | 1.000 | 1862 | tags=24%, list=14%, signal=28% |
| 327 | ATGTTTC,MIR-494 |  | 124 | -0.20 | -0.96 | 0.557 | 0.824 | 1.000 | 2389 | tags=22%, list=18%, signal=26% |
| 328 | CALRES\_RHESUS\_UP |  | 67 | -0.22 | -0.96 | 0.502 | 0.824 | 1.000 | 1943 | tags=25%, list=15%, signal=30% |
| 329 | ZHAN\_MULTIPLE\_MYELOMA\_VS\_NORMAL\_UP |  | 62 | -0.23 | -0.96 | 0.561 | 0.822 | 1.000 | 2155 | tags=26%, list=16%, signal=31% |
| 330 | BASSO\_HCL\_DIFF |  | 86 | -0.21 | -0.96 | 0.537 | 0.822 | 1.000 | 2440 | tags=24%, list=18%, signal=30% |
| 331 | HDACI\_COLON\_CUR\_UP |  | 99 | -0.21 | -0.96 | 0.546 | 0.826 | 1.000 | 1329 | tags=15%, list=10%, signal=17% |
| 332 | TTGGGAG,MIR-150 |  | 71 | -0.22 | -0.96 | 0.553 | 0.824 | 1.000 | 1214 | tags=15%, list=9%, signal=17% |
| 333 | BCNU\_GLIOMA\_MGMT\_48HRS\_DN |  | 157 | -0.19 | -0.96 | 0.597 | 0.828 | 1.000 | 1827 | tags=18%, list=14%, signal=20% |
| 334 | GCANCTGNY\_V$MYOD\_Q6 |  | 620 | -0.17 | -0.96 | 0.690 | 0.826 | 1.000 | 2112 | tags=17%, list=16%, signal=20% |
| 335 | V$HSF1\_01 |  | 176 | -0.19 | -0.96 | 0.633 | 0.826 | 1.000 | 1894 | tags=18%, list=14%, signal=20% |
| 336 | AGTCAGC,MIR-345 |  | 44 | -0.24 | -0.96 | 0.541 | 0.829 | 1.000 | 2649 | tags=32%, list=20%, signal=40% |
| 337 | NF90\_UP |  | 25 | -0.28 | -0.96 | 0.510 | 0.827 | 1.000 | 845 | tags=12%, list=6%, signal=13% |
| 338 | TTCCGTT,MIR-191 |  | 24 | -0.28 | -0.96 | 0.552 | 0.825 | 1.000 | 784 | tags=17%, list=6%, signal=18% |
| 339 | UVC\_TTD\_ALL\_UP |  | 76 | -0.21 | -0.95 | 0.528 | 0.827 | 1.000 | 2268 | tags=22%, list=17%, signal=27% |
| 340 | FRUCTOSE\_AND\_MANNOSE\_METABOLISM |  | 25 | -0.28 | -0.95 | 0.504 | 0.828 | 1.000 | 1366 | tags=24%, list=10%, signal=27% |
| 341 | V$CREB\_Q3 |  | 179 | -0.19 | -0.95 | 0.660 | 0.834 | 1.000 | 2250 | tags=22%, list=17%, signal=26% |
| 342 | V$SP1\_Q2\_01 |  | 179 | -0.19 | -0.95 | 0.612 | 0.835 | 1.000 | 2157 | tags=20%, list=16%, signal=23% |
| 343 | NF90\_DN |  | 38 | -0.25 | -0.95 | 0.543 | 0.843 | 1.000 | 887 | tags=18%, list=7%, signal=20% |
| 344 | GTGCAAA,MIR-507 |  | 101 | -0.21 | -0.94 | 0.605 | 0.849 | 1.000 | 2399 | tags=25%, list=18%, signal=30% |
| 345 | V$MYOGENIN\_Q6 |  | 171 | -0.19 | -0.94 | 0.647 | 0.846 | 1.000 | 1123 | tags=12%, list=8%, signal=13% |
| 346 | CHR7P13 |  | 29 | -0.26 | -0.94 | 0.511 | 0.849 | 1.000 | 826 | tags=10%, list=6%, signal=11% |
| 347 | UVC\_TTD-XPCS\_COMMON\_UP |  | 21 | -0.29 | -0.94 | 0.535 | 0.848 | 1.000 | 1448 | tags=19%, list=11%, signal=21% |
| 348 | SHIPP\_DLBCL\_CURED\_UP |  | 28 | -0.27 | -0.94 | 0.562 | 0.853 | 1.000 | 657 | tags=14%, list=5%, signal=15% |
| 349 | V$STAT1\_01 |  | 42 | -0.25 | -0.94 | 0.562 | 0.851 | 1.000 | 246 | tags=7%, list=2%, signal=7% |
| 350 | HDACI\_COLON\_SUL24HRS\_DN |  | 104 | -0.20 | -0.94 | 0.644 | 0.852 | 1.000 | 1282 | tags=15%, list=10%, signal=17% |
| 351 | HOGERKORP\_CD44\_DN |  | 21 | -0.28 | -0.94 | 0.577 | 0.850 | 1.000 | 1982 | tags=29%, list=15%, signal=34% |
| 352 | V$ARP1\_01 |  | 114 | -0.20 | -0.94 | 0.625 | 0.853 | 1.000 | 883 | tags=11%, list=7%, signal=11% |
| 353 | AGUIRRE\_PANCREAS\_CHR19 |  | 74 | -0.21 | -0.94 | 0.598 | 0.852 | 1.000 | 1790 | tags=16%, list=13%, signal=19% |
| 354 | TYROSINE\_METABOLISM |  | 28 | -0.27 | -0.93 | 0.576 | 0.856 | 1.000 | 2570 | tags=29%, list=19%, signal=35% |
| 355 | ZHAN\_MM\_CD1\_VS\_CD2\_UP |  | 69 | -0.22 | -0.93 | 0.582 | 0.855 | 1.000 | 1550 | tags=14%, list=12%, signal=16% |
| 356 | V$CETS1P54\_01 |  | 177 | -0.18 | -0.93 | 0.674 | 0.855 | 1.000 | 2267 | tags=22%, list=17%, signal=26% |
| 357 | CHR7P22 |  | 39 | -0.24 | -0.93 | 0.584 | 0.854 | 1.000 | 1965 | tags=18%, list=15%, signal=21% |
| 358 | CHR20Q11 |  | 68 | -0.22 | -0.93 | 0.606 | 0.853 | 1.000 | 2846 | tags=29%, list=21%, signal=37% |
| 359 | ACACTGG,MIR-199A,MIR-199B |  | 125 | -0.20 | -0.93 | 0.635 | 0.855 | 1.000 | 1462 | tags=13%, list=11%, signal=14% |
| 360 | CTAWWWATA\_V$RSRFC4\_Q2 |  | 260 | -0.18 | -0.93 | 0.771 | 0.854 | 1.000 | 2182 | tags=19%, list=16%, signal=23% |
| 361 | AD12\_ANY\_DN |  | 25 | -0.27 | -0.93 | 0.570 | 0.857 | 1.000 | 1818 | tags=24%, list=14%, signal=28% |
| 362 | V$EGR\_Q6 |  | 187 | -0.18 | -0.93 | 0.702 | 0.858 | 1.000 | 1694 | tags=17%, list=13%, signal=19% |
| 363 | SMITH\_HTERT\_DN |  | 61 | -0.22 | -0.93 | 0.629 | 0.856 | 1.000 | 2034 | tags=21%, list=15%, signal=25% |
| 364 | AGED\_MOUSE\_MUSCLE\_DN |  | 31 | -0.25 | -0.93 | 0.576 | 0.855 | 1.000 | 1433 | tags=16%, list=11%, signal=18% |
| 365 | RYTGCNWTGGNR\_UNKNOWN |  | 74 | -0.21 | -0.93 | 0.603 | 0.853 | 1.000 | 2520 | tags=27%, list=19%, signal=33% |
| 366 | V$ELK1\_01 |  | 166 | -0.19 | -0.93 | 0.688 | 0.854 | 1.000 | 2140 | tags=19%, list=16%, signal=23% |
| 367 | V$IRF\_Q6 |  | 163 | -0.19 | -0.92 | 0.695 | 0.857 | 1.000 | 2366 | tags=21%, list=18%, signal=26% |
| 368 | CHR8P11 |  | 32 | -0.25 | -0.92 | 0.603 | 0.857 | 1.000 | 1830 | tags=22%, list=14%, signal=25% |
| 369 | GACAGGG,MIR-339 |  | 53 | -0.22 | -0.92 | 0.627 | 0.859 | 1.000 | 2772 | tags=28%, list=21%, signal=36% |
| 370 | UVC\_XPCS\_ALL\_UP |  | 61 | -0.22 | -0.92 | 0.606 | 0.860 | 1.000 | 1588 | tags=18%, list=12%, signal=20% |
| 371 | CTGCAGY\_UNKNOWN |  | 524 | -0.17 | -0.92 | 0.841 | 0.866 | 1.000 | 2480 | tags=21%, list=19%, signal=25% |
| 372 | HPV31\_UP |  | 43 | -0.23 | -0.91 | 0.608 | 0.874 | 1.000 | 928 | tags=14%, list=7%, signal=15% |
| 373 | TAKEDA\_NUP8\_HOXA9\_8D\_UP |  | 121 | -0.19 | -0.91 | 0.673 | 0.873 | 1.000 | 1155 | tags=11%, list=9%, signal=12% |
| 374 | V$YY1\_02 |  | 172 | -0.18 | -0.91 | 0.688 | 0.871 | 1.000 | 1721 | tags=15%, list=13%, signal=17% |
| 375 | HDACI\_COLON\_TSABUT\_DN |  | 19 | -0.29 | -0.91 | 0.598 | 0.870 | 1.000 | 844 | tags=21%, list=6%, signal=22% |
| 376 | AGCTCCT,MIR-28 |  | 68 | -0.21 | -0.91 | 0.651 | 0.868 | 1.000 | 1747 | tags=19%, list=13%, signal=22% |
| 377 | UVC\_XPCS\_8HR\_UP |  | 58 | -0.22 | -0.91 | 0.658 | 0.876 | 1.000 | 1533 | tags=17%, list=12%, signal=19% |
| 378 | CHR20Q13 |  | 122 | -0.19 | -0.91 | 0.699 | 0.875 | 1.000 | 2926 | tags=30%, list=22%, signal=39% |
| 379 | SHEPARD\_BMYB\_MORPHOLINO\_UP |  | 137 | -0.19 | -0.91 | 0.736 | 0.874 | 1.000 | 1291 | tags=12%, list=10%, signal=14% |
| 380 | CHR4Q28 |  | 22 | -0.27 | -0.91 | 0.595 | 0.877 | 1.000 | 3814 | tags=45%, list=29%, signal=64% |
| 381 | CHR2Q36 |  | 24 | -0.26 | -0.91 | 0.578 | 0.876 | 1.000 | 2065 | tags=29%, list=16%, signal=34% |
| 382 | HDACI\_COLON\_CUR48HRS\_UP |  | 60 | -0.22 | -0.90 | 0.619 | 0.883 | 1.000 | 981 | tags=12%, list=7%, signal=13% |
| 383 | GCCATNTTG\_V$YY1\_Q6 |  | 306 | -0.17 | -0.90 | 0.866 | 0.885 | 1.000 | 2666 | tags=23%, list=20%, signal=28% |
| 384 | GRANDVAUX\_IRF3\_DN |  | 20 | -0.28 | -0.90 | 0.602 | 0.884 | 1.000 | 935 | tags=20%, list=7%, signal=21% |
| 385 | GLYCOGEN |  | 19 | -0.28 | -0.90 | 0.610 | 0.882 | 1.000 | 1227 | tags=16%, list=9%, signal=17% |
| 386 | RORIE\_ES\_PNET\_UP |  | 27 | -0.26 | -0.90 | 0.599 | 0.882 | 1.000 | 1444 | tags=22%, list=11%, signal=25% |
| 387 | CHR17P11 |  | 47 | -0.23 | -0.90 | 0.634 | 0.883 | 1.000 | 2696 | tags=23%, list=20%, signal=29% |
| 388 | V$HMEF2\_Q6 |  | 101 | -0.20 | -0.90 | 0.729 | 0.881 | 1.000 | 1063 | tags=12%, list=8%, signal=13% |
| 389 | RARRXRPATHWAY |  | 15 | -0.30 | -0.90 | 0.588 | 0.879 | 1.000 | 2350 | tags=27%, list=18%, signal=32% |
| 390 | V$OLF1\_01 |  | 178 | -0.18 | -0.89 | 0.795 | 0.894 | 1.000 | 1861 | tags=17%, list=14%, signal=19% |
| 391 | CHR5Q35 |  | 68 | -0.21 | -0.89 | 0.700 | 0.892 | 1.000 | 3090 | tags=31%, list=23%, signal=40% |
| 392 | ST\_DIFFERENTIATION\_PATHWAY\_IN\_PC12\_CELLS |  | 43 | -0.23 | -0.89 | 0.618 | 0.893 | 1.000 | 2542 | tags=28%, list=19%, signal=34% |
| 393 | TPA\_SKIN\_UP |  | 16 | -0.30 | -0.89 | 0.587 | 0.893 | 1.000 | 1789 | tags=19%, list=13%, signal=22% |
| 394 | CREB\_BRAIN\_2WKS\_UP |  | 21 | -0.27 | -0.89 | 0.630 | 0.892 | 1.000 | 816 | tags=14%, list=6%, signal=15% |
| 395 | GGCKCATGS\_UNKNOWN |  | 41 | -0.23 | -0.89 | 0.650 | 0.891 | 1.000 | 2736 | tags=29%, list=21%, signal=37% |
| 396 | V$RSRFC4\_Q2 |  | 154 | -0.18 | -0.89 | 0.770 | 0.891 | 1.000 | 2165 | tags=19%, list=16%, signal=22% |
| 397 | V$MYB\_Q3 |  | 169 | -0.18 | -0.89 | 0.808 | 0.891 | 1.000 | 1867 | tags=17%, list=14%, signal=20% |
| 398 | TAGGTCA,MIR-192,MIR-215 |  | 34 | -0.24 | -0.89 | 0.668 | 0.891 | 1.000 | 1806 | tags=26%, list=14%, signal=31% |
| 399 | PTENPATHWAY |  | 16 | -0.28 | -0.89 | 0.633 | 0.892 | 1.000 | 3115 | tags=38%, list=23%, signal=49% |
| 400 | TACAATC,MIR-508 |  | 52 | -0.21 | -0.88 | 0.703 | 0.893 | 1.000 | 681 | tags=10%, list=5%, signal=10% |
| 401 | V$IK1\_01 |  | 182 | -0.17 | -0.88 | 0.795 | 0.899 | 1.000 | 1226 | tags=12%, list=9%, signal=13% |
| 402 | ST\_JNK\_MAPK\_PATHWAY |  | 40 | -0.23 | -0.88 | 0.708 | 0.912 | 1.000 | 2345 | tags=23%, list=18%, signal=27% |
| 403 | GLYCOSPHINGOLIPID\_METABOLISM |  | 22 | -0.26 | -0.87 | 0.652 | 0.911 | 1.000 | 2053 | tags=23%, list=15%, signal=27% |
| 404 | V$RSRFC4\_01 |  | 177 | -0.17 | -0.87 | 0.848 | 0.910 | 1.000 | 1693 | tags=14%, list=13%, signal=16% |
| 405 | CHREBPPATHWAY |  | 16 | -0.29 | -0.87 | 0.616 | 0.911 | 1.000 | 835 | tags=13%, list=6%, signal=13% |
| 406 | V$NRF1\_Q6 |  | 161 | -0.18 | -0.87 | 0.821 | 0.910 | 1.000 | 2506 | tags=21%, list=19%, signal=26% |
| 407 | V$SMAD3\_Q6 |  | 162 | -0.18 | -0.87 | 0.854 | 0.917 | 1.000 | 1982 | tags=17%, list=15%, signal=19% |
| 408 | UVC\_HIGH\_ALL\_UP |  | 19 | -0.27 | -0.87 | 0.638 | 0.917 | 1.000 | 1816 | tags=21%, list=14%, signal=24% |
| 409 | V$LFA1\_Q6 |  | 168 | -0.17 | -0.87 | 0.820 | 0.916 | 1.000 | 1286 | tags=12%, list=10%, signal=13% |
| 410 | CCANNAGRKGGC\_UNKNOWN |  | 72 | -0.20 | -0.87 | 0.747 | 0.914 | 1.000 | 976 | tags=13%, list=7%, signal=13% |
| 411 | V$FOXO4\_02 |  | 175 | -0.17 | -0.87 | 0.880 | 0.917 | 1.000 | 859 | tags=10%, list=6%, signal=11% |
| 412 | INSULINPATHWAY |  | 21 | -0.27 | -0.87 | 0.652 | 0.915 | 1.000 | 1484 | tags=19%, list=11%, signal=21% |
| 413 | UVB\_NHEK3\_C6 |  | 29 | -0.25 | -0.86 | 0.700 | 0.920 | 1.000 | 2012 | tags=24%, list=15%, signal=28% |
| 414 | CHR10Q26 |  | 55 | -0.21 | -0.86 | 0.752 | 0.927 | 1.000 | 1895 | tags=16%, list=14%, signal=19% |
| 415 | LEE\_TCELLS9\_UP |  | 28 | -0.25 | -0.86 | 0.697 | 0.928 | 1.000 | 657 | tags=14%, list=5%, signal=15% |
| 416 | CMV\_UV-CMV\_COMMON\_HCMV\_6HRS\_UP |  | 20 | -0.26 | -0.85 | 0.679 | 0.933 | 1.000 | 1927 | tags=20%, list=14%, signal=23% |
| 417 | ZHAN\_MM\_CD138\_HP\_VS\_REST |  | 40 | -0.22 | -0.85 | 0.711 | 0.933 | 1.000 | 2032 | tags=25%, list=15%, signal=29% |
| 418 | VALINE\_LEUCINE\_AND\_ISOLEUCINE\_DEGRADATION |  | 35 | -0.23 | -0.85 | 0.737 | 0.936 | 1.000 | 3389 | tags=46%, list=25%, signal=61% |
| 419 | BUTANOATE\_METABOLISM |  | 27 | -0.24 | -0.85 | 0.746 | 0.939 | 1.000 | 2317 | tags=30%, list=17%, signal=36% |
| 420 | GH\_HYPOPHYSECTOMY\_RAT\_DN |  | 15 | -0.28 | -0.85 | 0.675 | 0.937 | 1.000 | 548 | tags=13%, list=4%, signal=14% |
| 421 | ANDROGEN\_GENES |  | 52 | -0.21 | -0.85 | 0.760 | 0.936 | 1.000 | 2077 | tags=21%, list=16%, signal=25% |
| 422 | IGF1PATHWAY |  | 20 | -0.27 | -0.85 | 0.707 | 0.936 | 1.000 | 1484 | tags=20%, list=11%, signal=22% |
| 423 | GGTAACC,MIR-409-5P |  | 29 | -0.23 | -0.84 | 0.725 | 0.939 | 1.000 | 423 | tags=10%, list=3%, signal=11% |
| 424 | ADIPOCYTE\_PPARG\_UP |  | 15 | -0.29 | -0.84 | 0.680 | 0.940 | 1.000 | 1717 | tags=27%, list=13%, signal=31% |
| 425 | TAAYNRNNTCC\_UNKNOWN |  | 112 | -0.18 | -0.84 | 0.840 | 0.938 | 1.000 | 1762 | tags=14%, list=13%, signal=16% |
| 426 | UVC\_LOW\_C3\_DN |  | 19 | -0.27 | -0.84 | 0.708 | 0.940 | 1.000 | 2823 | tags=37%, list=21%, signal=47% |
| 427 | PLATELET\_EXPRESSED |  | 33 | -0.23 | -0.84 | 0.758 | 0.939 | 1.000 | 2563 | tags=39%, list=19%, signal=49% |
| 428 | CHR13Q13 |  | 17 | -0.28 | -0.84 | 0.693 | 0.937 | 1.000 | 1510 | tags=24%, list=11%, signal=27% |
| 429 | TGFBETA\_C2\_UP |  | 18 | -0.27 | -0.84 | 0.688 | 0.937 | 1.000 | 2092 | tags=28%, list=16%, signal=33% |
| 430 | CCAWNWWNNNGGC\_UNKNOWN |  | 62 | -0.20 | -0.84 | 0.786 | 0.935 | 1.000 | 1644 | tags=16%, list=12%, signal=18% |
| 431 | ADIP\_VS\_FIBRO\_DN |  | 27 | -0.24 | -0.83 | 0.740 | 0.941 | 1.000 | 1645 | tags=22%, list=12%, signal=25% |
| 432 | ABRAHAM\_MM\_VS\_AL\_DN |  | 22 | -0.25 | -0.83 | 0.725 | 0.942 | 1.000 | 1929 | tags=27%, list=14%, signal=32% |
| 433 | CARM\_ERPATHWAY |  | 26 | -0.24 | -0.83 | 0.730 | 0.941 | 1.000 | 2350 | tags=27%, list=18%, signal=33% |
| 434 | CHR17Q12 |  | 44 | -0.21 | -0.83 | 0.798 | 0.945 | 1.000 | 3033 | tags=30%, list=23%, signal=38% |
| 435 | V$SMAD4\_Q6 |  | 168 | -0.17 | -0.83 | 0.911 | 0.943 | 1.000 | 2154 | tags=17%, list=16%, signal=20% |
| 436 | ROSS\_PML\_RAR |  | 80 | -0.19 | -0.83 | 0.841 | 0.946 | 1.000 | 1940 | tags=20%, list=15%, signal=23% |
| 437 | AGED\_MOUSE\_NEOCORTEX\_DN |  | 50 | -0.21 | -0.82 | 0.779 | 0.947 | 1.000 | 859 | tags=12%, list=6%, signal=13% |
| 438 | MUNSHI\_MM\_UP |  | 65 | -0.19 | -0.82 | 0.783 | 0.945 | 1.000 | 970 | tags=11%, list=7%, signal=12% |
| 439 | CMV\_HCMV\_6HRS\_DN |  | 55 | -0.20 | -0.82 | 0.827 | 0.945 | 1.000 | 2907 | tags=33%, list=22%, signal=42% |
| 440 | YU\_CMYC\_DN |  | 39 | -0.21 | -0.82 | 0.759 | 0.948 | 1.000 | 1518 | tags=13%, list=11%, signal=14% |
| 441 | PYK2PATHWAY |  | 27 | -0.23 | -0.82 | 0.784 | 0.948 | 1.000 | 1936 | tags=22%, list=15%, signal=26% |
| 442 | V$COUP\_DR1\_Q6 |  | 169 | -0.16 | -0.81 | 0.949 | 0.953 | 1.000 | 795 | tags=8%, list=6%, signal=9% |
| 443 | ABRAHAM\_AL\_VS\_MM\_UP |  | 23 | -0.25 | -0.81 | 0.755 | 0.953 | 1.000 | 1929 | tags=26%, list=14%, signal=30% |
| 444 | V$SREBP1\_Q6 |  | 182 | -0.16 | -0.81 | 0.970 | 0.958 | 1.000 | 1741 | tags=15%, list=13%, signal=17% |
| 445 | AGTCTAG,MIR-151 |  | 20 | -0.25 | -0.81 | 0.775 | 0.958 | 1.000 | 1276 | tags=15%, list=10%, signal=17% |
| 446 | GCTGAGT,MIR-512-5P |  | 39 | -0.21 | -0.80 | 0.807 | 0.963 | 1.000 | 2821 | tags=26%, list=21%, signal=32% |
| 447 | MOUSE\_TISSUE\_TESTIS |  | 26 | -0.24 | -0.80 | 0.755 | 0.964 | 1.000 | 2012 | tags=27%, list=15%, signal=32% |
| 448 | CAGCCTC,MIR-485-5P |  | 101 | -0.17 | -0.80 | 0.898 | 0.963 | 1.000 | 2641 | tags=27%, list=20%, signal=33% |
| 449 | CMV\_HCMV\_TIMECOURSE\_18HRS\_DN |  | 21 | -0.24 | -0.80 | 0.790 | 0.966 | 1.000 | 2585 | tags=33%, list=19%, signal=41% |
| 450 | UVB\_NHEK4\_6HRS\_UP |  | 27 | -0.23 | -0.79 | 0.782 | 0.969 | 1.000 | 2750 | tags=41%, list=21%, signal=51% |
| 451 | P53HYPOXIAPATHWAY |  | 19 | -0.25 | -0.79 | 0.781 | 0.967 | 1.000 | 3180 | tags=42%, list=24%, signal=55% |
| 452 | CMV\_HCMV\_TIMECOURSE\_8HRS\_UP |  | 20 | -0.25 | -0.79 | 0.791 | 0.971 | 1.000 | 2425 | tags=30%, list=18%, signal=37% |
| 453 | VEGF\_MMMEC\_12HRS\_UP |  | 27 | -0.23 | -0.79 | 0.801 | 0.970 | 1.000 | 2737 | tags=33%, list=21%, signal=42% |
| 454 | CHR7P14 |  | 28 | -0.23 | -0.79 | 0.801 | 0.970 | 1.000 | 3331 | tags=32%, list=25%, signal=43% |
| 455 | GH\_EXOGENOUS\_EARLY\_UP |  | 24 | -0.23 | -0.79 | 0.770 | 0.970 | 1.000 | 335 | tags=8%, list=3%, signal=9% |
| 456 | CCAGGGG,MIR-331 |  | 66 | -0.19 | -0.78 | 0.905 | 0.970 | 1.000 | 1079 | tags=11%, list=8%, signal=11% |
| 457 | V$LXR\_Q3 |  | 61 | -0.19 | -0.78 | 0.863 | 0.969 | 1.000 | 2248 | tags=21%, list=17%, signal=26% |
| 458 | PITX2PATHWAY |  | 16 | -0.26 | -0.78 | 0.772 | 0.967 | 1.000 | 9827 | tags=100%, list=74%, signal=381% |
| 459 | BIOGENIC\_AMINE\_SYNTHESIS |  | 15 | -0.26 | -0.78 | 0.793 | 0.966 | 1.000 | 9837 | tags=100%, list=74%, signal=382% |
| 460 | GENOTOXINS\_24HRS\_DISCR |  | 35 | -0.21 | -0.78 | 0.830 | 0.967 | 1.000 | 1431 | tags=17%, list=11%, signal=19% |
| 461 | SIG\_IL4RECEPTOR\_IN\_B\_LYPHOCYTES |  | 27 | -0.22 | -0.78 | 0.842 | 0.969 | 1.000 | 2419 | tags=26%, list=18%, signal=32% |
| 462 | DAC\_FIBRO\_UP |  | 17 | -0.25 | -0.78 | 0.776 | 0.968 | 1.000 | 1400 | tags=18%, list=11%, signal=20% |
| 463 | CHR12Q23 |  | 42 | -0.20 | -0.78 | 0.867 | 0.967 | 1.000 | 3240 | tags=36%, list=24%, signal=47% |
| 464 | RASPATHWAY |  | 22 | -0.23 | -0.77 | 0.783 | 0.971 | 1.000 | 3332 | tags=41%, list=25%, signal=54% |
| 465 | CCTGAGT,MIR-510 |  | 33 | -0.21 | -0.77 | 0.823 | 0.972 | 1.000 | 2820 | tags=33%, list=21%, signal=42% |
| 466 | CHR11P15 |  | 161 | -0.15 | -0.76 | 0.979 | 0.973 | 1.000 | 3365 | tags=30%, list=25%, signal=40% |
| 467 | V$LXR\_DR4\_Q3 |  | 62 | -0.18 | -0.76 | 0.940 | 0.973 | 1.000 | 1530 | tags=15%, list=11%, signal=16% |
| 468 | GGCNKCCATNK\_UNKNOWN |  | 86 | -0.17 | -0.76 | 0.949 | 0.973 | 1.000 | 1721 | tags=17%, list=13%, signal=20% |
| 469 | ARAPPATHWAY |  | 20 | -0.23 | -0.76 | 0.801 | 0.972 | 1.000 | 1963 | tags=25%, list=15%, signal=29% |
| 470 | SPRYPATHWAY |  | 17 | -0.25 | -0.76 | 0.805 | 0.974 | 1.000 | 1484 | tags=24%, list=11%, signal=26% |
| 471 | CHR3P24 |  | 24 | -0.22 | -0.75 | 0.838 | 0.977 | 1.000 | 2957 | tags=29%, list=22%, signal=37% |
| 472 | V$CDP\_01 |  | 60 | -0.18 | -0.75 | 0.933 | 0.978 | 1.000 | 859 | tags=10%, list=6%, signal=11% |
| 473 | CATRRAGC\_UNKNOWN |  | 91 | -0.16 | -0.75 | 0.962 | 0.978 | 1.000 | 1062 | tags=11%, list=8%, signal=12% |
| 474 | V$E47\_02 |  | 165 | -0.15 | -0.74 | 0.983 | 0.981 | 1.000 | 2810 | tags=22%, list=21%, signal=28% |
| 475 | PASSERINI\_OXIDATION |  | 19 | -0.24 | -0.74 | 0.846 | 0.980 | 1.000 | 1774 | tags=21%, list=13%, signal=24% |
| 476 | YGCANTGCR\_UNKNOWN |  | 94 | -0.16 | -0.73 | 0.966 | 0.984 | 1.000 | 3253 | tags=30%, list=24%, signal=39% |
| 477 | TSA\_CD4\_UP |  | 25 | -0.21 | -0.73 | 0.880 | 0.987 | 1.000 | 1894 | tags=16%, list=14%, signal=19% |
| 478 | GCRPATHWAY |  | 17 | -0.23 | -0.72 | 0.862 | 0.990 | 1.000 | 3162 | tags=41%, list=24%, signal=54% |
| 479 | BETA\_ALANINE\_METABOLISM |  | 26 | -0.21 | -0.72 | 0.906 | 0.988 | 1.000 | 2086 | tags=23%, list=16%, signal=27% |
| 480 | TCRPATHWAY |  | 43 | -0.19 | -0.72 | 0.938 | 0.987 | 1.000 | 1795 | tags=19%, list=13%, signal=21% |
| 481 | CTCTAGA,MIR-526C,MIR-518F,MIR-526A |  | 41 | -0.19 | -0.72 | 0.932 | 0.988 | 1.000 | 2505 | tags=24%, list=19%, signal=30% |
| 482 | CALRES\_MOUSE\_DN |  | 39 | -0.19 | -0.71 | 0.954 | 0.992 | 1.000 | 2910 | tags=26%, list=22%, signal=33% |
| 483 | AAGGGAT,MIR-188 |  | 57 | -0.17 | -0.70 | 0.957 | 0.995 | 1.000 | 1806 | tags=18%, list=14%, signal=20% |
| 484 | HDACI\_COLON\_SUL12HRS\_UP |  | 20 | -0.21 | -0.70 | 0.884 | 0.994 | 1.000 | 1086 | tags=15%, list=8%, signal=16% |
| 485 | GGTGTGT,MIR-329 |  | 78 | -0.16 | -0.70 | 0.955 | 0.993 | 1.000 | 2149 | tags=17%, list=16%, signal=20% |
| 486 | TSA\_RKO\_UP |  | 17 | -0.23 | -0.69 | 0.896 | 0.996 | 1.000 | 1824 | tags=24%, list=14%, signal=27% |
| 487 | GLYCOGEN\_METABOLISM |  | 33 | -0.19 | -0.69 | 0.946 | 0.995 | 1.000 | 1292 | tags=12%, list=10%, signal=13% |
| 488 | FMLPPATHWAY |  | 34 | -0.18 | -0.68 | 0.953 | 0.997 | 1.000 | 1936 | tags=21%, list=15%, signal=24% |
| 489 | HBX\_HCC\_DN |  | 22 | -0.20 | -0.67 | 0.925 | 0.999 | 1.000 | 1826 | tags=18%, list=14%, signal=21% |
| 490 | UV\_ESR\_OLD\_UNREG |  | 16 | -0.21 | -0.65 | 0.949 | 1.000 | 1.000 | 231 | tags=6%, list=2%, signal=6% |
| 491 | CHR19P12 |  | 16 | -0.21 | -0.65 | 0.898 | 1.000 | 1.000 | 3006 | tags=38%, list=23%, signal=48% |
| 492 | CALRES\_MOUSE\_UP |  | 28 | -0.18 | -0.65 | 0.952 | 1.000 | 1.000 | 1717 | tags=18%, list=13%, signal=20% |
| 493 | IFNA\_UV-CMV\_COMMON\_HCMV\_6HRS\_UP |  | 29 | -0.18 | -0.65 | 0.962 | 1.000 | 1.000 | 1733 | tags=17%, list=13%, signal=20% |
| 494 | ARGININE\_AND\_PROLINE\_METABOLISM |  | 41 | -0.16 | -0.64 | 0.986 | 1.000 | 1.000 | 3090 | tags=32%, list=23%, signal=41% |
| 495 | KIM\_TH\_CELLS\_UP |  | 44 | -0.17 | -0.64 | 0.981 | 1.000 | 1.000 | 2274 | tags=20%, list=17%, signal=25% |
| 496 | NI2\_LUNG\_DN |  | 19 | -0.19 | -0.62 | 0.965 | 1.000 | 1.000 | 10753 | tags=100%, list=81%, signal=518% |
| 497 | NICK\_RHAPC\_UP |  | 32 | -0.17 | -0.61 | 0.970 | 1.000 | 1.000 | 1315 | tags=13%, list=10%, signal=14% |
| 498 | CK1PATHWAY |  | 15 | -0.20 | -0.61 | 0.927 | 1.000 | 1.000 | 10626 | tags=100%, list=80%, signal=494% |
| 499 | YTTCCNNNGGAMR\_UNKNOWN |  | 30 | -0.17 | -0.60 | 0.983 | 1.000 | 1.000 | 802 | tags=10%, list=6%, signal=11% |
| 500 | NOS1PATHWAY |  | 21 | -0.18 | -0.60 | 0.968 | 1.000 | 1.000 | 226 | tags=5%, list=2%, signal=5% |
| 501 | ACAWNRNSRCGG\_UNKNOWN |  | 50 | -0.15 | -0.60 | 0.997 | 1.000 | 1.000 | 3530 | tags=38%, list=26%, signal=52% |
| 502 | 41BBPATHWAY |  | 18 | -0.18 | -0.59 | 0.968 | 1.000 | 1.000 | 1326 | tags=11%, list=10%, signal=12% |
| 503 | AGED\_MOUSE\_RETINA\_ANY\_UP |  | 20 | -0.18 | -0.58 | 0.974 | 1.000 | 1.000 | 2738 | tags=25%, list=21%, signal=31% |
| 504 | PGC1APATHWAY |  | 23 | -0.16 | -0.52 | 0.994 | 1.000 | 1.000 | 226 | tags=4%, list=2%, signal=4% |
| 505 | UCALPAINPATHWAY |  | 15 | -0.17 | -0.51 | 0.995 | 1.000 | 1.000 | 1795 | tags=13%, list=13%, signal=15% |
| 506 | CHR5Q34 |  | 20 | -0.15 | -0.49 | 0.992 | 1.000 | 1.000 | 2832 | tags=25%, list=21%, signal=32% |
| 507 | NFATPATHWAY |  | 50 | -0.11 | -0.46 | 1.000 | 1.000 | 1.000 | 3162 | tags=24%, list=24%, signal=31% |
| 508 | EICOSANOID\_SYNTHESIS |  | 17 | -0.13 | -0.40 | 1.000 | 1.000 | 1.000 | 3757 | tags=35%, list=28%, signal=49% |
Table: Gene sets enriched in phenotype **na **[plain text format]****
